# Bronchiectasis diagnosed after renal transplantation: a retrospective multicenter study

**DOI:** 10.1186/s12890-015-0133-9

**Published:** 2015-11-07

**Authors:** Sandra Dury, Charlotte Colosio, Isabelle Etienne, Dany Anglicheau, Elodie Merieau, Sophie Caillard, Joseph Rivalan, Eric Thervet, Marie Essig, François Babinet, Jean-François Subra, Olivier Toubas, Philippe Rieu, Claire Launois, Jeanne-Marie Perotin-Collard, François Lebargy, Gaëtan Deslée, on behalf of the Spiesser group

**Affiliations:** Service des Maladies Respiratoires, Hôpital Maison Blanche, CHU, 45, rue de Cognacq-Jay, 51092 Reims, Cedex France; EA 4683 Université de Médecine et de Pharmacie, Reims, France; Service de Néphrologie, Hôpital Maison Blanche, CHU, Reims, France; Service de Néphrologie, Rouen University Hospital, Rouen, France; Service de Néphrologie, Hôpital Necker, APHP, Paris, France; Service de Néphrologie, CHU, Tours, France; Service de Néphrologie, Hôpitaux Universitaires, Strasbourg, France; Service de Néphrologie, CHU, Rennes, France; Service de Néphrologie, Hôpital Européen Georges Pompidou, APHP, Paris, France; Service de Néphrologie, CHU, Limoges, France; Néphrologie-Dialyse ECHO - Pôle Santé Sud, Le Mans, France; Service de Néphrologie, CHU, Angers, France; Service de Radiologie, Hôpital Maison Blanche, CHU, Reims, France; Unité 903 Inserm, Reims, France

**Keywords:** Bronchiectasis, Renal transplantation, CT scan, Mycophenolic acid, Immunosuppression

## Abstract

**Background:**

Bronchiectasis is characterized by abnormal, permanent and irreversible dilatation of the bronchi, usually responsible for daily symptoms and frequent respiratory complications. Many causes have been identified, but only limited data are available concerning the association between bronchiectasis and renal transplantation.

**Methods:**

We conducted a retrospective multicenter study of cases of bronchiectasis diagnosed after renal transplantation in 14 renal transplantation departments (French SPIESSER group). Demographic, clinical, laboratory and CT scan data were collected.

**Results:**

Forty-six patients were included (mean age 58.2 years, 52.2 % men). Autosomal dominant polycystic kidney disease (32.6 %) was the main underlying renal disease. Chronic cough and sputum (50.0 %) were the major symptoms leading to chest CT scan. Mean duration of symptoms before diagnosis was 1.5 years [0–12.1 years]. Microorganisms were identified in 22 patients, predominantly *Haemophilus influenzae*. Hypogammaglobulinemia was observed in 46.9 % patients. Bronchiectasis was usually extensive (84.8 %). The total bronchiectasis score was 7.4 ± 5.5 with a significant gradient from apex to bases. Many patients remained symptomatic (43.5 %) and/or presented recurrent respiratory tract infections (37.0 %) during follow-up. Six deaths (13 %) occurred during follow-up, but none were attributable to bronchiectasis.

**Conclusions:**

These results highlight that the diagnosis of bronchiectasis should be considered in patients with *de novo* respiratory symptoms after renal transplantation. Further studies are needed to more clearly understand the mechanisms underlying bronchiectasis in this setting.

## Background

Renal transplantation is the most common form of solid organ transplantation. Acute and chronic rejections are the most dreaded complications. The main immunosuppressive drugs used to control allograft rejection include corticosteroids, calcineurin inhibitors (cyclosporine A, tacrolimus) and inhibitors of T- and B-cell proliferation (mycophenolic acid (MPA) including mycophenolate mofetil (MMF) and mycophenolate sodium). The most common adverse effect of these immunosuppressive drugs is increased susceptibility to infections, mainly lung infections [1].

Bronchiectasis is an acquired anatomical disorder characterized by permanent and irreversible abnormal dilatation of the bronchi [2]. Chronic productive cough, recurrent exacerbations, pneumonia and hemoptysis are the main symptoms. High-resolution computed tomography (HRCT) is required to establish the diagnosis [3]. Many causes of bronchiectasis have been identified, such as postinfectious, congenital or underlying anatomical or systemic disease [4, 5]. Of note, neither renal transplantation nor immunosuppressive drugs are usually considered to be potential causes of bronchiectasis. Several studies have recently reported the development of bronchiectasis in adults [6, 7] and children [8–10] after renal transplantation and hypothesized that MPA, used since 1996, might be a causative agent.

The objectives of this study were to analyze the characteristics of a series of cases of bronchiectasis observed after renal transplantation and to evaluate potential associations.

## Methods

### Study protocol

This retrospective multicenter study was conducted in 14 renal transplantation departments participating in the French SPIESSER group. Each center was asked to identify patients with bronchiectasis occurring after renal transplantation. Nine centers (Reims, Angers, Limoges, Hôpital Européen Georges Pompidou in Paris, Hôpital Necker in Paris, Rennes, Rouen, Strasbourg, Tours) identified eligible patients with bronchiectasis diagnosed after renal transplantation from 1982 to 2014.

**Fig. 1:**
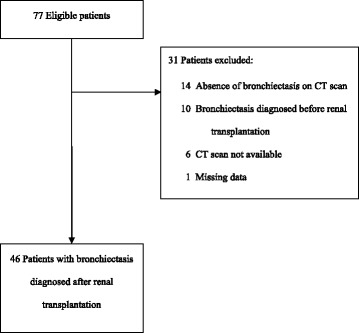
Flowchart of patient selection for the study

Inclusion criteria were a diagnosis of bronchiectasis after renal transplantation, and age 18 years or older. Exclusion criteria were bronchiectasis diagnosed before renal transplantation, no available CT scan for confirmation of the diagnosis or very incomplete clinical data (Fig. 1). The ethical approval was obtained multi-centre. The authorization to access patient data was obtained from the French Advisory Committee for Data Processing in Health Research (CCTIRS, Comité Consultatif sur le Traitement de l’Information en matière de Recherche dans le domaine de la Santé) (n°13.018) and approved by the national commission for the personal data protection (CNIL, Comité National de l’Informatique et des Libertés) (n° 913412). The collection and analyses of the data were fully anonymised.

A standard form was used to collect demographic, clinical and laboratory data from each patient’s medical records. Obstructive disorder was defined by FEV_1_/FVC < 0.7 before bronchodilatators.

### CT scan

Each CT scan was reviewed by a panel of pulmonologists (SD, FL, GD) and chest radiologist (OT) with a final consensus interpretation. All CT scans reviewed were performed with the patient in the supine position at end-inspiratory volume using multidetector CT scanners. One- to 5- mm-thick slices at 5- to 10-mm intervals were analyzed from the lung apices to the lung bases. The diagnosis of bronchiectasis was defined by an airway lumen inner diameter greater than the diameter of the accompanying pulmonary artery, absence of tapering of bronchi, and visualization of bronchi within 1 cm of the pleural surface [3]. As proposed by Diederich et al., the extent of bronchiectasis was classified into five categories on a lobar basis [11], and a progressive score was used to quantify bronchiectasis in each lobe: no involvement (0), less than 25 % involvement (1), 26–50 % involvement (2), 51–75 % involvement (3) and greater than 75 % involvement (4) resulting in a maximum score of 24 per patient. The lingula was considered to be a separate lobe for this analysis. The distribution of bronchiectasis was defined as follows: localized (one lobe affected) or extensive (at least two lobes affected). The type of bronchiectasis was assessed as cylindrical, varicose or cystic [3]. Other CT findings related to bronchiectasis were recorded: pulmonary consolidations, bronchiolar nodules, mucoid impactions and pulmonary atelectasis. When multiple CT scans were performed following the diagnosis of bronchiectasis, all scans were assessed but only the most recent scan was selected for long-term follow-up analysis. The longitudinal changes in CT-scan were defined as follows: worsening (subsequent score of bronchiectasis greater than initial score), stabilization (subsequent score identical to initial score) and improvement (subsequent score less than initial score).

### Statistical analysis

Statistical analysis was performed using EpiInfo statistical software (version 3.2.2). Data are expressed as mean ± standard deviation [minimum, maximum]. Comparisons were performed with Fisher’s exact test and Student *t* test. A *p* value < 0.05 was considered significant.

## Results and discussion

Forty-six of the 77 eligible patients were included in the study (Fig. 1) from Reims (*n* = 18), Rouen (*n* = 9), Hôpital Necker in Paris (*n* = 8), Tours (*n* = 4), Strasbourg (*n* = 3), Angers (*n* = 1), Rennes (*n* = 1), Hôpital Européen Georges Pompidou in Paris (*n* = 1) and Limoges (*n* = 1).

### Patient characteristics

Clinical characteristics at the time of diagnosis of bronchiectasis are summarized in Table 1. The mean age was 58.2 years. Twenty-four patients (52.2 %) were men. Chronic cough and sputum (50 %) were the main symptoms leading to chest CT scan. The mean duration of symptoms before diagnosis was 1.5 years. Most patients had undergone only one renal transplantation (89.1 %). Autosomal dominant polycystic kidney disease (ADPKD) (32.6 %) was the main underlying renal disease. Immunosuppressive drugs used prior to the diagnosis of bronchiectasis are listed in Table 1. All patients received steroids and all but three of the patients received MPA (93.5 %).

**Table 1:** Demographic and Clinical Characteristics at the Time of Diagnosis

Variables	Total (*n* = 46)
Male	24 (52.2)
Age (years)	58.2 ± 13.6 [20.4–82.1]
Duration of symptoms before diagnosis (years)	1.5 ± 2.3 [0–12.1]
Presenting symptoms	
Cough and sputum > 3 months	23 (50.0)
Hemoptysis	2 (4.3)
Single respiratory tract infection	14 (30.4)
Recurrent bronchitis	16 (34.8)
Recurrent pneumonia	9 (19.6)
Incidental	3 (6.5)
Interval between first renal transplantation and first symptoms (years)	9.1 ± 7.3 (0.2–25.3)
Interval between first renal transplantation and diagnosis (years)	10.9 ± 7.5 (0.4–32.0)
Cause of end-stage renal disease	
Chronic glomerulonephritis	10 (21.7)
Diabetic nephropathy	0 (0)
Autosomal dominant polycystic kidney disease	15 (32.6)
Chronic tubulointerstitial nephritis	11 (23.9)
Unknown cause of end-stage renal disease	4 (8.7)
Other renal disease	6 (13.0)
Age at the first renal transplantation (years)	47.3 ± 15.1 [9.4–72.9]
Number of renal transplantations	
One renal transplantation	41 (89.1)
Two renal transplantations	4 (8.7)
Three renal transplantations	1 (2.2)
Immunosuppressive regimen	
Induction therapy	
Anti-lymphocyte globulins	21 (45.7)
Basiliximab	8 (17.4)
Steroids	46 (100)
Purine synthesis inhibitor	
Mycophenolic acid (MPA)	43 (93.5)
Azathioprine	14 (30.4)
Calcineurin inhibitors	
Cyclosporine	32 (69.6)
Tacrolimus	15 (32.5)
mTOR inhibitors	
Sirolimus	12 (26.1)
Everolimus	1 (2.2)
Rituximab	1 (2.2)

Data are expressed as frequency (percentage) or mean ± standard deviation [minimum, maximum]

Few patients exhibited respiratory symptoms before renal transplantation, including chronic cough (*n* = 3), dyspnea (*n* = 2), annual bronchitis (*n* = 2) and pneumonia (*n* = 1). No patient exhibited chronic sputum. Eighteen patients (40.9 %) were current or former smokers and two had a diagnosis of chronic obstructive pulmonary disease. Significant pre-existing extrarenal medical conditions were unusual, including mycobacterial infection (*n* = 1) and rheumatoid arthritis (*n* = 1) (Table 2). No case of chronic respiratory bacterial colonization was identified.

**Table 2:** Pulmonary Status and Comorbidities before Renal Transplantation

Variables	Total (*n* = 46)
Respiratory symptom	
Chronic cough	3 (6.5)
Chronic sputum	0 (0)
Dyspnea	2 (4.3)
Respiratory tract infection	3 (6.5)
Annual bronchitis	2 (4.3)
Pneumonia	1 (2.2)
Smoker (current or former)^a^	18 (40.9)
Pack-years	19.4 ± 10.2 (5–40)
Chronic obstructive pulmonary disease	2 (4.3)
Asthma	1 (2.2)
Mycobacterial infection	1 (2.2)
Comorbid illnesses	
Gastroesophageal reflux disease	17 (37.0)
Sinusitis	8 (17.4)
Rheumatoid arthritis	1 (2.2)

Data are expressed as frequency (percentage) or mean ± standard deviation [minimum, maximum]

^a^Missing data for 2 patients

### CT scans

Bronchiectases were cylindrical (100 %) and usually extensive (84.8 %). Table 3 describes the characteristics of the chest CT scan. The mean total bronchiectasis score was 7.4 ± 5.5 with a significant gradient from apex to bases. Bronchiolar nodules (41.3 %) and mucoid impactions (21.7 %) were the most common associated CT scan findings.

**Table 3:** CT scan features at the Time of Diagnosis

Variables	Score (*n* = 46)
Distribution of bronchiectasis	
Localized	7 (15.2)
Extensive	39 (84.8)
Total bronchiectasis score	7.4 ± 5.5 [1–24]
Right upper lobe	1 ± 1 [0–4]
Middle lobe	1.2 ± 1.4 [0–4]
Right lower lobe	1.6 ± 1.4 [0–4]
Culmen	0.7 ± 1.0 [0–4]
Lingula	1.0 ± 1.0 [0–4]
Left lower lobe	1.9 ± 1.5 [0–4]
Associated signs	
Mucoid impactions	10 (21.7)
Pulmonary atelectasis	4 (8.7)
Bronchiolar nodules	19 (41.3)
Pulmonary consolidations	2 (4.3)

Data are expressed as frequency (percentage) or mean ± standard deviation [minimum, maximum]

Chest X-ray prior to renal transplantation was available for 36 patients and was considered to be normal. Chest CT scan was performed before (*n* = 3) or just after (*n* = 9) renal transplantation in 12 patients and demonstrated no signs of bronchiectasis.

### Spirometry, laboratory, and microbiological data

Spirometry, laboratory and microbiological examinations were performed inconstantly at the time of diagnosis of bronchiectasis (Table 4). For patients with available data (*n* = 36), mean lymphocyte count was 1295 cells/mm^3^. Hypogammaglobulinemia, defined as gammaglobulins less than 9 g/dL and/or IgG less than 7 g/L, was observed in 15 patients (46.9 %) associated with lymphopenia in all but one case. Mean MPA area under the concentration-time curve (AUC) evaluated in 21 patients was 42.1 mg.h/L for a mean MPA daily dose of 1.5 g/day. Spirometry was available for 22 patients and showed an obstructive disorder in 8 cases (36.4 %). Microbiological data were obtained in 34 cases from sputum analyses (*n* = 4) or fiberoptic bronchoscopy procedure (*n* = 30). Microorganisms were identified in 22 cases. *Haemophilus influenzae* was the most common pathogen. Concomitant pathogens were associated with *Haemophilus influenzae* in 7 patients, including *Streptococcus species* (*n* = 2), *Aspergillus fumigatus* (*n* = 2), *Escherichia coli* (*n* = 2), and *Pseudomonas aeruginosa* (*n* = 1) (data not shown).

**Table 4:** Laboratory, Microbiology, Spirometry Data at the time of the Diagnosis of Bronchiectasis

Variables	Total (*n* = 46)
Serum creatinine (μmol/L)	155.9 ± 83.5 [63.0–602.0]
Creatinine clearance (mL/min, MDRD formula)	46.4 ± 25.6 [17.0–173.0]
Lymphocytes (/mm^3^) (*n* = 36)	1295 ± 717 [19–3200]
B Lymphocytes (*n* = 19)	182 ± 296 [1–1194]
T Lymphocytes (*n* = 19)	781 ± 646 [17–2029]
CD4+ T Lymphocytes (*n* = 19)	562 ± 446 [9–1786]
CD8+ T Lymphocytes (*n* = 19)	352 ± 296 [8–1287]
Electrophoresis (*n* = 21)	
Gammaglobulins (g/L)	8.0 ± 2.7 [4.0–14.0]
Immunoglobulin assays (*n* = 18)	
IgG (g/L)	8.2 ± 3.2 [3.2–15.5]
IgG1 (g/L) (*n* = 8)	5.6 ± 3.4 [0–11.1]
IgG2 (g/L) (*n* = 8)	2.0 ± 1.3 [0–4.1]
IgG3 (g/L) (*n* = 8)	0.3 ± 0.1 [0–0.5]
IgG4 (g/L) (*n* = 8)	0.2 ± 0.3 [0–0.8]
IgA (g/L)	1.4 ± 0.8 [0.2–3.5]
IgM (g/L)	0.6 ± 0.4 [0.1–1.4]
Hypogammaglobulinemia^a^	15 (46.9)
MPA treatment at time of diagnosis	42 (91.3)
MPA daily dose (g) (*n* = 41)	1.5 ± 0.6 [0.5–3.5]
MPA area under the curve (*n* = 21, mg.h/L)	42.1 ± 15.4 [20.5–80.0]
Spirometry (*n* = 22)	
Obstructive disorder	8 (36.4)
Microbiological data (*n* = 34)	
Sputum	4
Fiberoptic Bronchoscopy	30
Positive microbiological findings	22 (64.7)
* Haemophilus influenzae*	12
*Streptococcus species*	3
*Pseudomonas aeruginosa*	5
*Methicillin-resistant Staphylococcus aureus*	1
*Aspergillus fumigatus*	2
Other bacteria	4

Data are expressed as frequency (percentage) or mean ± standard deviation [minimum, maximum]

^a^Defined as gammaglobulins less than 9 g/dL and/or IgG less than 7 g/L, and available for 32 patients either by electrophoresis and/or immunoglobulin assay

### Initial management and outcome

MPA was stopped at the time of diagnosis of bronchiectasis in 3 patients and during follow-up in another 8 patients. Immunoglobulin replacement therapy was initiated in 6 patients either at the time of diagnosis (*n* = 3) or during follow-up (*n* = 3). The main symptoms reported after the diagnosis of bronchiectasis were chronic sputum (43.5 %) and recurrent respiratory tract infections (37.0 %). None of the six deaths (13 %) was attributable to bronchiectasis (Table 5).

**Table 5:** Follow-up Data

Variables	Total (*n* = 46)
Medical decision at the time of diagnosis	
MPA withdrawal	3
Immunoglobulin replacement therapy	3
Follow-up after diagnosis (months)	36.3 ± 33.3 [1–150]
Cough and chronic sputum	20 (43.5)
Respiratory tract infections	17 (37.0)
Number of episodes of acute bronchitis per year	0.2 ± 0.5 [0–2]
Number of episodes of pneumonitis per year	0.07 ± 0.2 [0–0.6]
Subsequent medical decision (*n* = 45)	
Unchanged	35
MPA withdrawal	8
Immunoglobulin replacement therapy	3
Death	6 (13 %)
Interval between diagnosis of bronchiectasis and death (years)	5.3 ± 4.4 [1.2–11.0]
Respiratory causes	0 (0)

Data are expressed as frequency (percentage) or mean ± standard deviation [minimum, maximum]

### Longitudinal changes in CT scan

At least one chest CT scan was performed after the diagnosis of bronchiectasis in 28 patients (60.9 %). The last CT scan available for each patient was performed 2.6 ± 2.5 years after the diagnosis of bronchiectasis. A trend towards worsening of the total bronchiectasis score was observed at follow-up for these 28 patients (7.7 ± 6.2 *vs.* 10.9 ± 6.7; *p* = 0.07). Bronchiectasis was more extensive in 19 patients (67.9 %), while stabilization was observed in 3 patients (10.7 %) and improvement was observed in 6 patients (21.4 %). Bronchiolar nodules were more frequent (*p* = 0.01).

### Discussion

To our knowledge, this study reports the largest series of bronchiectasis diagnosed after renal transplantation. The main finding of this study is that the clinical and microbiological characteristics of bronchiectasis are similar to those usually described in non-cystic fibrosis (CF) bronchiectasis [12]: (1) chronic sputum or recurrent bronchitis are the most common symptoms; (2) an obstructive disorder is frequently associated; (3) *Haemophilus influenzae* is the most common micro-organism identified. However, some results need to be highlighted. First, bronchiectasis can be diagnosed very late after renal transplantation, as illustrated by the two previous reports in adults with a mean time to diagnosis ranging from 3 to 11.7 years [6, 7]. Second, a CT scan gradient was observed from the apex to the bases. Previous reports have described a predominance of bronchiectasis in lower lobes in 3 patients, while the distribution of bronchiectasis was not specified in the other 12 cases [6–8]. Interestingly, other forms of non-CF bronchiectasis also exhibit such predominance in the bases [13, 14]. Third, the bronchiectasis score frequently deteriorated during follow-up. As observed in our study, some previous reports have shown that patients with non-CF bronchiectasis had persistent or worsening symptoms on long-term follow-up [15], but very limited data are available on the course of bronchiectasis based on follow-up CT scan score [16, 17]. Finally, the overall mortality in our series was 13 % with a median follow-up of 3 years, similar to the results of recent studies that have reported mortality rates ranging between 16.3 % at 4 years and 29.7 % at 13 years in non-CF bronchiectasis [18–20]. In a long-term prospective study, Loebinger et al. found that the primary cause of death was respiratory (70.4 %), especially respiratory infection or failure [19]. In contrast, despite frequent respiratory infections, no death was related to a respiratory cause in our series.

Prior to the study by Pijnenburg et al. in 2004, no case of bronchiectasis occurring after renal transplantation had been reported in the literature [8]. In our study, only two patients had a diagnosis of bronchiectasis before 2004 (data not shown). Bronchiectases were then mainly diagnosed within the ten last years, which may be related to expanded indications of CT-scan and changes in immunosuppression strategy. The absence of systematic chest CT scan before or just after renal transplantation does not allow pre-existing bronchiectasis to be formally excluded. A chest CT scan without bronchiectasis was available for only 12 patients (three before and nine after renal transplantation). The presence of asymptomatic bronchiectasis before transplantation in some cases therefore cannot be formally excluded. In particular, one patient had rheumatoid arthritis and another had a history of mycobacterial infection, two potential causes of bronchiectasis [12, 21]. Moreover, patients with ADPKD could also be at increased risk of bronchiectasis, as recent studies have demonstrated an increased prevalence of mild-to-moderate cylindrical bronchiectasis with bilateral lower lung predominance in ADPKD [22–24]. ADPKD is associated with defective primary ciliary function in renal epithelial cells. Functional abnormalities in polycystin-1 and 2, two membrane regulatory proteins expressed in the cilia of both human airway epithelial and airway smooth muscle cells, may result in radiological bronchiectasis due to decreased mucociliary clearance or impaired airway injury repair [22, 24]. However, these studies present a number of limitations including the absence of data on comorbidities associated with bronchiectasis [22] and the inclusion of transplant recipients [23]. At least, the minimal interval 0.4 year between first renal transplantation and diagnosis of bronchiectasis, probably too short to develop bronchiectasis, argues for undetected pre-existing bronchiectasis.

Bronchiectasis can be induced by primary or secondary immunodeficiency [12, 21, 25, 26]. Drug-induced immunosuppression following transplantation predisposes to recurrent lung infections and increases the risk of bronchiectasis after bone marrow, heart or lung transplantation [27–29]. Low immunoglobulin and mannose binding protein levels after renal transplantation are associated with infectious complications [30]. In our series, 15 patients exhibited hypogammaglobulinemia, which may contribute to the development of bronchiectasis [7].

Three recent studies have suggested that MPA may be a causative factor of bronchiectasis [6–8]. MPA is a relatively new immunosuppressive drug commonly used in renal transplantation for the prevention and treatment of allograft rejection. According to the results of these three studies, the potential involvement of MPA is based on the following arguments: 1) some patients did not have any respiratory symptoms despite long periods with other immunosuppressive drugs [8]; 2) an improvement of symptoms after MPA withdrawal was observed in some patients [6]; 3) some cases of bronchiectasis have been described in children who have received renal transplantation with MPA, whereas bronchiectasis is usually very uncommon at this age in the absence of CF or immunodeficiency [8]. In our series, five patients not treated by MPA for a first renal transplantation developed symptomatic bronchiectasis after a second or third renal transplantation with MPA treatment. Two mechanisms have been proposed to explain the role of MPA in bronchiectasis. First, MPA, as a potent inosine monophosphate dehydrogenase inhibitor, inhibits purine synthesis and severely depresses both cell-mediated and humoral immunity by inhibiting T- and B-cell proliferation. The resulting hypogammaglobulinemia has been shown to be more frequent and more severe in patients receiving MPA compared to other immunosuppressive drugs [31]. Only six of the 15 patients with hypogammaglobulinemia in our study received immunoglobulin replacement therapy. Second, MPA may directly affect bronchial epithelium by altering mucociliary clearance [32].

Our results should be interpreted in the context of the several limitations of this study. 1) The retrospective identification of the cases of bronchiectasis revealed after renal transplantation is a major limitation. Even if the study design was as rigorous possible, we can not exclude that a significant number of cases have not been identified by the centres. 2) The retrospective design of this study does not allow estimating the prevalence of the disease. 3) Thoracic CT scans were analysed in all the cases by a panel of pulmonologists, and radiologist. Cases were included in the study in presence of defined bronchiectasis. However, since HRCT scan was not available for each patients, we cannot rule out that distal bronchiectasis should not be visualized in some patients. 4) Observation bias may exist, even if it has been minimized by using a standardized data collection form. In particular, information on respiratory medical history may have been underreported. 5) The spirometry, biological and microbiological data were very heterogeneous. Moreover, the role of potentially pathogenic micro-organisms in the clinical course of bronchiectasis cannot be determined in absence of repeated respiratory samplings, micro-organisms quantification and characterisation of associated clinical status. 6) No conclusion can be drawn on the interest of MPA withdrawal or immunoglobulin replacement therapy due to the retrospective design and the small number of patients. Despite these limitations, we believe that our study provides important findings on the characteristics of bronchiectasis occurring after renal transplantation.

Finally, it should be stressed that, even after intensive investigation, one or more causative factors are identified in only 47 % of cases of bronchiectasis [21]. It can therefore be hypothesized that multifactorial mechanisms, including underlying diseases, immunosuppressive drugs and respiratory tract infections, could contribute to the pathogenesis or clinical emergence of bronchiectasis after renal transplantation. Due to the retrospective design of our study and the small number of patients, we were not able to perform subgroup analyses in order to determine the burden of causal variables such as ADPKD, MPA and immunoglobulin deficiency. Similar studies would be interesting in other solid organ (liver and heart) transplantation.

## Conclusion

These results highlight the fact that the diagnosis of bronchiectasis and the practice of a chest CT-scan should be considered in renal transplant recipients exhibiting respiratory symptoms such as cough and recurrent respiratory tract infections. The pathophysiological mechanisms remain to be elucidated and require further studies. In particular, the role of MPA, the long-term course, and the therapeutic management of bronchiectasis in this setting have yet to be defined.

## Abbreviations

ADPKD: Autosomal dominant polycystic kidney disease
CF: Cystic fibrosis
HRCT: High-resolution computed tomography
MMF: Mycophenolate mofetil
MPA: Mycophenolic acid
